# Attractiveness and Specificity of Different Polyethylene Blue Screens on *Stomoxys calcitrans* (Diptera: Muscidae)

**DOI:** 10.3390/insects11090575

**Published:** 2020-08-27

**Authors:** Shukri Sharif, Emmanuel Liénard, Gérard Duvallet, Lucas Etienne, Clément Mongellaz, Christelle Grisez, Michel Franc, Emilie Bouhsira, Philippe Jacquiet

**Affiliations:** 1 UMR INRAE ENVT 1225, Interactions Hôtes Agents Pathogènes, Université de Toulouse, 31076 Toulouse, France; shukri.sharif@envt.fr (S.S.); emmanuel.lienard@envt.fr (E.L.); clement.mongellaz@hotmail.fr (C.M.); christelle.grisez@envt.fr (C.G.); emilie.bouhsira@envt.fr (E.B.); 2 CEFE, Université de Montpellier, CNRS, EPHE, IRD, Université Paul Valéry Montpellier 3, 34000 Montpellier, France; gduvallet@aol.com (G.D.); lucasetienne.34@gmail.com (L.E.); 3 Laboratoire de Parasitologie, École Nationale Vétérinaire de Toulouse, 31076 Toulouse, France; michel.franc@envt.fr

**Keywords:** stable fly, trapping, control, visual target

## Abstract

**Simple Summary:**

The haematophagous fly *Stomoxys calcitrans* is considered as a major pest of livestock worldwide. Insecticides have been extensively used to control this pest but resistance to these chemical compounds is now reported in many countries. Therefore, a more sustainable and efficient control is needed. New blue polyethylene sticky screens have been proved to be very attractant for stable flies. They are produced at a lower price than all blue fabric screens or traps. More than 70% of flies are captured on the lower half (30 to 60 cm above ground) of the screens. In our conditions, very few non-target fauna was captured as very few pollinators were caught by these screens. These results are highlighting the interest of these blue polyethylene screens to control stable flies in cattle farms, in comparison with more expensive blue fabrics.

**Abstract:**

*Stomoxys calcitrans* is considered as a major pest of livestock worldwide. Insecticides have been extensively used to control this pest but resistance to these chemical compounds is now reported in many countries. Therefore, a more sustainable and efficient control is needed. Seven different types of blue screens, with reflectances around 460 nm, were tested during summer 2016 in southwestern France to evaluate their attractiveness and their specificity for stable flies. Height of the screen and orientation (east or west) of a blue screen were also considered. High levels of *S. calcitrans* captures were recorded during this study (from 141 to 7301 individuals per blue screen and per day) whereas the numbers of tabanids and pollinator insects remained extremely low (less than 10 individuals per screen and per day). No significant difference in attractiveness has been shown between the different types of blue screens. The lower half of the blue screens caught significantly more stable flies (70%) than the higher half (30%). The “east” side of the screen attracted 60% of stable flies but this was not significantly different from the west side. These results are highlighting the interest in these blue polyethylene screens for controlling stable flies in cattle farms, in comparison with more expensive blue fabrics.

## 1. Introduction

The stable fly, *Stomoxys calcitrans* (Linnaeus, 1758) is a cosmopolitan pest of livestock, which compromises animal and human welfare. Males and females are harassing hematophagous insects, feeding preferentially on ungulates but also on dogs and humans, and cause painful bites [1,2]. High levels of biting activity lead to substantial reductions in beef cattle weight gains and in milk production in dairy cows [3,4,5,6,7,8]. The stable fly is also a mechanical and a biological vector of numerous pathogens of veterinary and zoonotic importance [9].

The control of stable flies relies mainly on the use of insecticides applied on beef or dairy cattle. The exposure of stable flies to insecticides, applied on cattle, is limited by their feeding behavior: they spend most of their time off the host, the complete blood meal is achieved in less than 3 min, and the preferential bite sites are legs of cattle and horses where insecticide concentrations are the lowest [10]. However, insecticides could also be applied on walls of barns where stable flies spend most of their time, thus increasing the selective pressure on stable fly populations. Indeed, resistance to pyrethroids has been reported recently in Florida [11,12], and in southwestern France [13], and probably occurs in many other parts of the world [14,15]. Alternative and sustainable control methods that minimize the use of insecticides in fly control management are needed to address the spread of resistance and the increasing public concern over effects of insecticides on health and environment.

Trapping is considered as an appropriate method to monitor population dynamics of hematophagous flies and could readily help in the control of some of them such as tsetse flies [16]. Methods of biting-fly trapping used for tsetse flies in Africa were subsequently tested for stable flies. Blue fabric traps such as Nzi traps [17] and Vavoua traps [18] are useful to monitor stable flies’ populations [19]. Vavoua traps have been used in different studies of seasonal dynamics of populations in the Indian Ocean [20], Thailand [21] and in mainland France [22]. However, the total numbers of stable flies entering the Vavoua traps per day are relatively low in each of these studies (most of the time less than 100 stable flies per trap and per day even at the seasonal peak of activity), except in La Reunion Island where Gilles et al. (2007) [19] reported that one Vavoua trap could catch up to 600 individuals per day. This could be due to a lack of attractiveness of the trap itself and/or to the behavior of the stable flies around the traps. Indeed, only a low percentage (less than 25%) of the insects that landed on traps finally entered them [17]. This observation suggests that an insecticide-impregnated screen used as a resting site would kill much more insects than a trap itself. This strategy was proposed a long time ago [23,24] for tsetse flies and was successfully employed in some Gambian sleeping sickness foci [16]. However, before implementing a control strategy with attractive screens, their specificity should be carefully checked. A non-specific one, catching fewer biting flies than pollinating insects or in equal numbers for example, will be discarded immediately from an integrated control strategy.

Therefore, the objectives of this study were:

(i) To compare the trapping efficiency/attractiveness towards *S. calcitrans* of several types of blue screen designs, with reflectances around 460 nm, and by their composition. Visual stimuli are of high importance in the detection of hosts by hematophagous flies [25]. Electroretinographic recordings of stable flies showed strong peaks of visual activities occurring at 330–360 nm, 460–525 nm and 605–635 nm [26,27]. In the study of Zhu et al., 2016 [27], young stable flies were more responsive to white, whereas gravid females preferred blue.
(ii) To study potential biases in the trapping efficiency (height of screens, east or west exposed sites of a blue screen, proximity of different hosts and manure sites etc.).
(iii) To study the specificity of attractiveness of the different blue screens tested.

## 2. Materials and Methods

### 2.1. Study Sites

**Figure 1:**
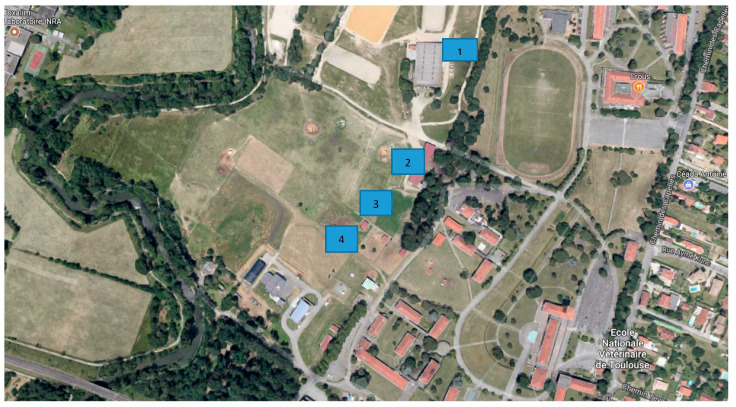
Zone of study and traps locations: site 1—horse manure, site 2—horse paddock 1, site 3— horse paddock 2, site 4—sheep paddock (Google map modified).

The study was conducted on the campus of the National Veterinary School of Toulouse (NVST), southwestern France (43.36° North and 1.43° East, 189 m a.s.l.). One quarter of the NVST property (a total of 53 hectares) is composed of pasturelands, which are equally divided into horse and cattle/sheep paddocks. Four locations were chosen to represent different types of micro-environments on the NVST campus. The locations used in this study were selected due to the high stable fly activity observed each year [22]. They were located close to horse manure (site 1), two different horse paddocks (sites 2 and 3) and a cattle/sheep paddock (site 4) (Figure 1). Screens were placed along the perimeters (and not inside the paddocks), 50 m apart to ensure that the attractive qualities of one screen were not influenced by those of another screen at this distance. Horses, sheep, and cattle were the main animals present in and near the area of study.

### 2.2. Blue Screens

**Figure 2:**
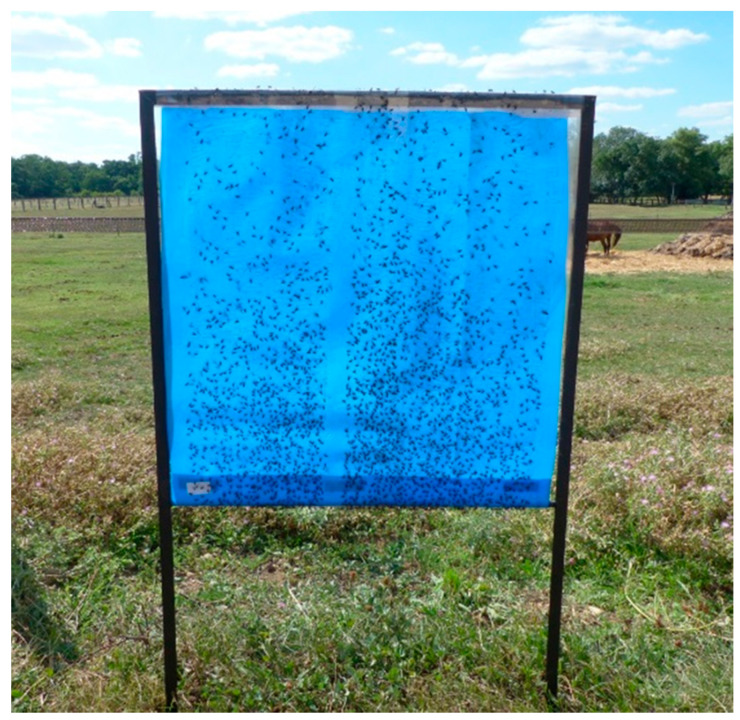
Metal frame and blue screen; the picture was taken at 16: 30 p.m. i.e., 8 h after the blue screen installation. At the back side: horse and manure.

Seven types of blue screens (60 cm × 60 cm of size) fixed on metal frames (Figure 2) were compared and named as follows: polyethylene blue screens (KLM1, KLM2, KLM3, KLM4, Sample1, Sample2) obtained from A to Z Textile Mills Ltd., and one polyester fabric blue screen (“Burma”, CR solon N°41, from Chai Rung Textile ltd, Bangkok, Thailand). The latter was used as “reference” in two different experiments. The reflectance of the different blue screens was measured by A to Z Textile Mills Ltd. in Arusha. All the screens showed a peak of reflectance around 460 nm and the percentage of reflectance was between 40 and 65%. The blue polyethylene screens had a higher percentage of reflectance. These devices are under a patent in progress. The fabric for the Burma screen was obtained from Thailand where it is readily available in the market. Both sides of each blue screen were covered by a sticky transparent film (Luminos 4 adhesive Rolls, ref FE217, Rentokill Initial Supplies, Liverpool, UK) to retain all attracted insects landing on the screens. As the Luminos adhesive rolls are 30 cm wide, 2 strips of sticky films were rolled over the blue screens (Figure 2). For each trapping day, blue screens were installed in the morning (8:30 a.m.) and removed in the afternoon (4:30 p.m.) making an eight-hour trapping session, as the activity of *Stomoxys calcitrans* is known to be diurnal in southwestern France [22]. The traps were installed so that the bases of the screens were at 30 cm above ground. Blue screens were installed to provide an “east” side and a “west” side for each screen. At each collection date, all flies captured on the sticky films were divided into three categories (stable flies, tabanids and pollinating insects) and counted trap by trap. The numbers of flies caught on the lower half and on the upper half of a blue screen were recorded. In addition, as both sides of a blue screen were used for trapping insects, the number of stable flies caught on the west side and on the east side of the blue screen were recorded. Male/female ratios were not evaluated.

### 2.3. Experimental Design

The study was run for six weeks from 26 July 2016 to 5 September 2016. Two different experiments were conducted: experiment one (12 days of trapping during three weeks from 26 July to 12 August) with the comparison of KLM2, KLM3, KLM4 and Burma blue screens and experiment two (12 days of trapping during three weeks from 16 August to 5 September) with the comparison of KLM1, Sample 1, Sample 2 and Burma blue screens. Burma blue screen was used all around the study to compare the number of stable flies caught in the two experiments.

Each trapping week included four days of trapping. The catches of each blue screen were compared using a 4 × 4 Latin square design (four types of traps at four locations over 4 days). This was repeated three times (multiple Latin squares with a total of 12 trapping days) in each experiment.

### 2.4. Statistical Analyses

Data were analyzed using Shapiro–Wilk test to verify the normality. Following a normal distribution, a variance analysis and multiple comparison tests of the means were performed (RStudio version 3.3.3; packages “agricolae” and “pgirmess”). Comparisons between the distribution numbers of *S. calcitrans* (up/down and west/east modalities) on the blue screen were performed using Student’s *t*-test. All *p*-values were considered significant at ≤0.05. If the normality distribution was not verified, the difference or similarity of the stable fly numbers was checked by using a Kruskal–Wallis test.

## 3. Results

**Figure 3:**
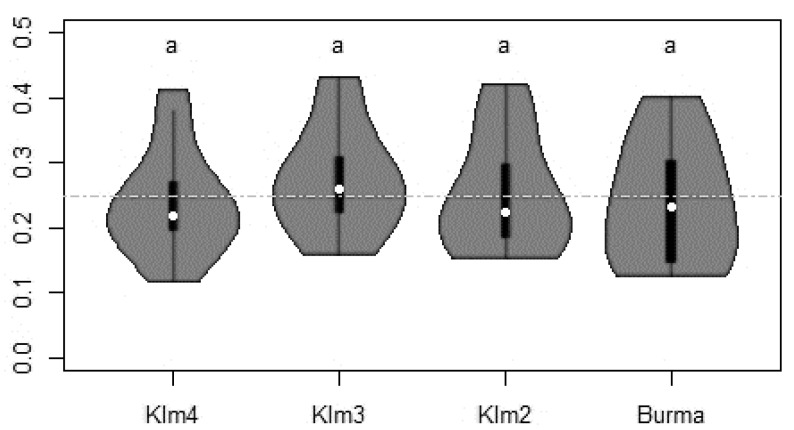
Proportions of stable flies captured by different blue screens during the experiment 1 (26 July to 12 August). The total numbers of stable flies caught per trap were: 30,476 (KLM2); 34,107 (KLM3); 30,298 (KLM4) and 30,523 (Burma). Inside each «violin plot», the white dot represents the median, the black vertical bar represents the first quartile below, the third quartile above. The lower and upper adjacent values (black line stretched from the bar = whiskers) are defined, respectively, as: the first quartile − 1.5 × interquartile and the third quartile + 1.5 × interquartile. The shape around the outside is the sampling distribution using a kernel estimator (density plot of the variable). When it is large, there are more data at this value. A horizontal grey line at 0.25 has been added.

In experiment 1, a total of 125,404 stable flies were caught (26 July to 12 August). No significant difference in attractiveness towards stable flies was recorded between the four different types of traps: Klm2, Klm3, Klm4 and Burma blue screens caught 30,476 (24.3%), 34,107 (27.2%), 30,298 (24.2%) and 30,523 (24.3%) flies, respectively. This homogeneity of stable fly captures between the different types of blue screens was observed during the three weeks of trapping whatever the total number of flies caught per week (Figure 3).

**Figure 4:**
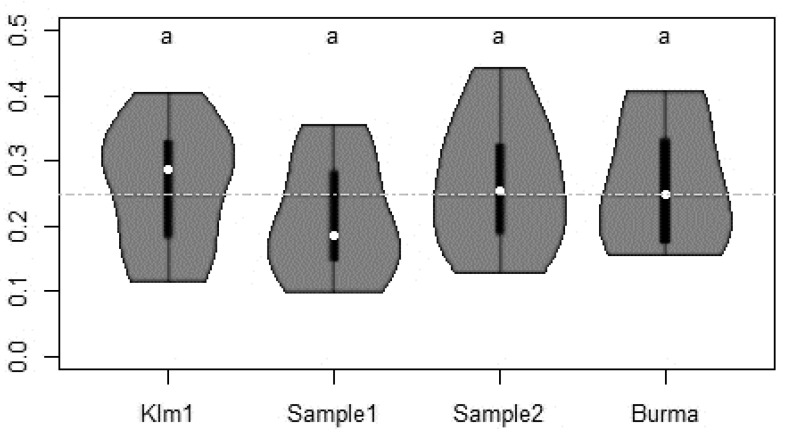
Proportion of stable flies captured by different blue screens during the experiment 2 (16 August to 5 September). The total numbers of stable flies caught per trap were: 9641 (KLM1); 8356 (Sample 1); 10,592 (Sample 2) and 10,699 (Burma). See Figure 3 for explanations about the violin plots.

In experiment 2 (16 August to 5 September), a total of 39,288 stable flies were caught during the 12 days of trapping which represents about 31% of the total number of *S. calcitrans* caught during the first experiment. As in experiment 1, no significant differences in attractiveness to stable flies were observed between the four different types of screens tested (Klm1, Sample 1, Sample 2, and Burma blue screens) (Figure 4).

Significantly higher numbers of stable flies (69 to 82%) were captured on the lower half of the screens compared to the upper half, whatever the experiment, the type of blue screen and the replication.

The east side of the blue screens captured about 60% of the total number of stable flies in the experiment 1 and about 56% of the total number of stable flies in experiment 2. This was observed whatever the type of blue screen and the repetition (Table 1). However, the difference between both sides (east versus west) was not significant.

**Figure 5:**
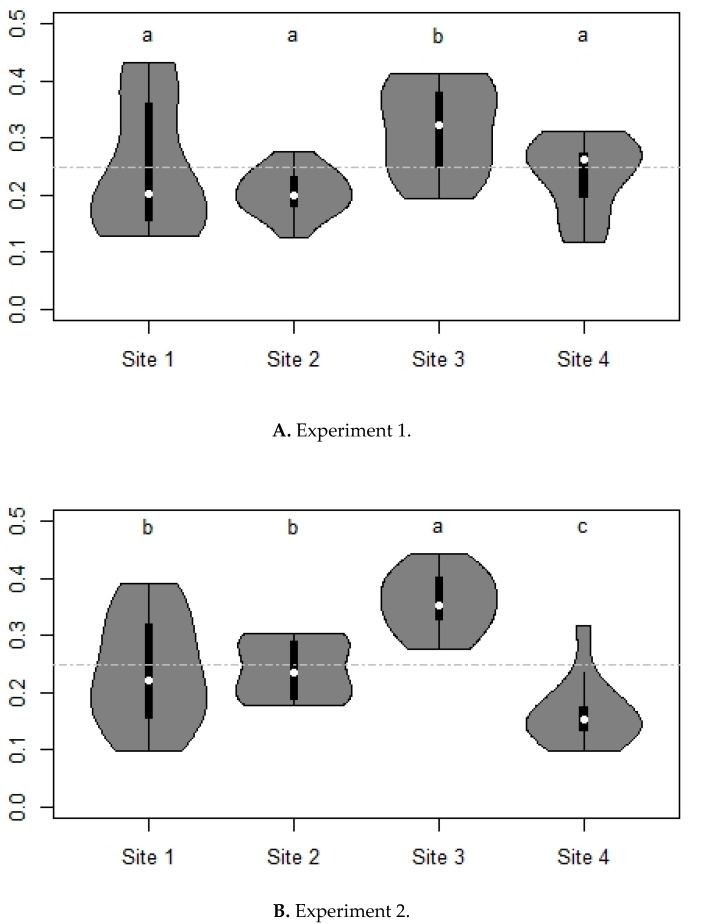
Proportion of stable flies captured by location in experiment 1 (**A**) and in experiment 2 (**B**). See Figure 3 for explanations about the violin plots.

Regarding the influence of screen’s location, site 3 (horse paddock 2) showed a significantly higher number of *S. calcitrans* captures in both experiments compared to the three other sites (Figure 5).

Pollinating insects and horse flies were caught in very low numbers during the two experiments: 32 and 20 individuals, respectively, for experiment 1 and 74 and 8 individuals, respectively, for experiment 2, whatever the type of blue screen or the repetition (Table 2). These two categories of insects represented 0.04% and 0.2% of the total number of captures during experiments 1 and 2, respectively. It should be noted that the total number of pollinating insects caught in experiment 2 was 2.3 times higher than in experiment 1 and that Tabanid captures decreased from 20 individuals in experiment 1 to 8 individuals in experiment 2. Most of these Tabanids belonged to the *Haematopota* genus and a few to the *Tabanus* according to Chlava et al. (1972) [28].

## 4. Discussion

The results showed high efficacies of trapping with high numbers of stable fly captures during trapping sessions lasting only eight hours (8.30 a.m. to 4.30 p.m.). The total number of stable flies ranged from 141 to 7301 individuals per trap per day. These levels of capture are substantially higher than those reported by Taylor and Berkebile (2006) [2] in their comparison between five adhesive traps and the Nzi trap, and Gilles et al. (2007) [19] who compared two Alsynite sticky traps and two phthalogen blue fabric traps (Vavoua and Nzi). Jacquiet et al. (2014) [22] observed a maximum of 120 stable flies per Vavoua trap and per day during the summer months on the NVST campus. This latest difference in levels of capture observed in the same environment could be due to highly favorable conditions for stable fly activities during summer 2016 and/or to a remarkably high attractiveness of the blue screen devices tested in this study. Another important difference resides in the fact that stable flies could land on fabric traps (such as Vavoua traps) without entering the trap, leading to an underestimation of the total number of attracted flies [29]. Beresford and Sutcliffe (2017) [30] reported that live stable flies caught on sticky traps could inhibit additional stable flies from being captured. Regarding the number of stable flies caught and the density of these flies on the small surface of our blue screens, this phenomenon probably did not occur in the conditions of the NVST campus and with the tested blue screens. The coverage of the screens with a plastic sticky film could have influenced the attractivity of the screens, giving a homogeneous appearance to all surfaces. This has probably an influence, but in many other experiments using the same methodology to compare different blue fabrics, we observed differences between the different fabrics [31]. In addition, the main objective of the present study was to evaluate the efficacy of polyethylene screens in comparison to a polyester fabric. This polyester fabric has already been compared to other different fabrics in another experiment [32].

Several studies have been conducted to understand the relationships between the height of the traps and the efficacy of catching of stable flies. Beresford and Sutcliffe (2008) [33] found that 30–60 cm is the optimum height of the sticky traps in the field, considering the height of the grass. Black and Krafsur (1985) [34] demonstrated that stable flies preferred lower perching sites either when they were flying near the host searching the blood meal or when they were resting. Showler and Osbrink (2015) [35] confirmed that in the field, stable flies can fly about 90 cm above the ground in their local movements near the host. Our results showed also that the most attractive part of the blue screen was the lowest part (between 30 and 60 cm above the ground). It was not possible to sex the *S. calcitrans* captured by blue screens in this study due to the very high number of flies caught and that were tightly adhering onto the sticky film (making them difficult to sample for observation under a binocular microscope). However, in a complementary study in 2017, we observed that the sex ratio was almost 1 to 1 (47% of males and 53% of females on 150 stable flies examined) under the same sampling conditions.

The east and the west exposed sides of the blue screen caught slightly different numbers of stable flies without significant difference. The east side caught a little bit more than the west side and this was observed whatever the type of blue screen and the repetition. This might suggest once again that activity of stable flies was more important in the morning (when the east side of the blue screen is facing the sun) than in the afternoon during the period of both experiments. Our results also showed that the specificity of attractiveness was remarkably high as demonstrated by the low numbers of pollinators captured by the different types of blue screens. As sticky traps are expendable products and cannot be recycled or re-used, it is quite difficult to imagine the systematic association between blue screens and sticky films in a sustainable and cost-effective stable fly control program. Therefore, the impregnation of the blue screen with an insecticide in a combination of “specific attraction” and “contact mortality” could be an alternative solution to the topical application of such insecticides on cattle. Nevertheless, the landing time of stable flies on the blue screens and the mortality rates following short contacts between the screens and the flies should be carefully checked.

## 5. Conclusions

The most striking result of this study was the high numbers of *S. calcitrans* caught during both experiments whatever the type of blue screens or the repetition. In most instances, more than a thousand stable flies were caught by one blue screen in only 8 h. This is indicative of extremely high population numbers with a uniform distribution and the similarity in attraction and capture among the various traps.

The second important finding of this study was that the different devices tested in both experiments showed the same attractiveness for stable flies on the NVST campus. This similarity could have been influenced by the plastic sticky film placed on screen surfaces. But this suggests that polyethylene films could be used instead of fabrics for the control of hematophagous insects using insecticide-impregnated screens, consequently at a much lower price. Beside this, the results obtained in this study showed clearly that most stable flies tend to land on the lower half of the blue screen, i.e., between 30 and 60 cm height above the ground level.

## Figures and Tables

**Table 1:** Total numbers of *Stomoxys calcitrans* caught during three sessions of 4 days of trapping according to the east/west sides of the blue screens. In red: percentages of *S. calcitrans* caught in the east side of a blue screen/total number of stable flies caught on both sides during the entire experiment (total of 12 days of trapping/experiment).

**Experiment 1**										
**Type of Blue Screen/Latin Square**	**Klm2**		**Klm3**		**Klm4**		**Burma**		**Total**	
	**East**	**West**	**East**	**West**	**East**	**West**	**East**	**West**	**East**	**West**
Latin square 1	5294	3922	5762	4520	4927	3515	4715	3182	20,698	15,139
Latin square 2	8154	6136	10,129	6834	9190	6300	9189	6322	36,662	25,592
Latin square 3	4571	2399	4157	2705	4176	2190	4140	2975	17,044	10,269
Total	17,019	12,457	20,048	14,059	18,293	12,005	18,044	12,479	74,404	51,000
	59%		59%		60%		59%		59.3%	
**Experiment 2**										
**Type of Blue Screen/Latin Square**	**Klm1**		**Sample 1**		**Sample 2**		**Burma**		**Total**	
	**East**	**West**	**East**	**West**	**East**	**West**	**East**	**West**	**East**	**West**
Latin square 1	2622	1638	1756	1396	3160	2460	1963	2060	9501	7554
Latin square 2	1190	1128	1170	676	966	731	1448	1048	4774	3583
Latin square 3	2031	1032	1480	1878	2054	1221	2048	2132	7613	6263
Total	5843	3798	4406	3950	6180	4412	5459	5240	21,888	17,400
	60.6%		52.7%		58.3%		51%		55.7%	

**Table 2:** Total numbers of pollinating insects (including bees and syrphids) and horse flies caught during three sessions of 4 days of trapping/experiment.

**Experiment 1**										
**Type of Blue Screen** **Latin Square**	**Klm2** **Pollinators/Tabanids**		**Klm3** **Pollinators/Tabanids**		**Klm4** **Pollinators/Tabanids**		**Burma** **Pollinators/Tabanids**		**Total** **Pollinators/Tabanids**	
Latin square 1	10	0	1	0	1	0	1	0	13	0
Latin square 2	3	7	3	2	1	2	0	2	7	13
Latin square 3	5	3	3	2	3	1	1	1	12	7
Total	18	10	7	4	5	3	2	3	32	20
**Experiment 2**										
**Type of Blue Screen** **Latin Square**	**Klm1** **Pollinators/Tabanids**		**Sample 1** **Pollinators/Tabanids**		**Sample 2** **Pollinators/Tabanids**		**Burma** **Pollinators/Tabanids**		**Total** **Pollinators/Tabanids**	
Latin square 1	12	0	5	2	8	1	7	2	32	5
Latin square 2	6	1	5	1	6	1	4	0	21	3
Latin square 3	5	0	5	0	7	0	4	0	21	0
Total	23	1	15	3	21	2	15	2	74	8
